# Supplementary material for: Strain-insensitive viscoelastic perovskite film for intrinsically stretchable neuromorphic vision-adaptive transistors
Source: Nat Commun. 2024 Apr 10;15:3123. doi: 10.1038/s41467-024-47532-w (PMC11006893; doi:10.1038/s41467-024-47532-w)
Supplement: Supplementary file 1 — Supporting Information [file 41467_2024_47532_MOESM1_ESM.pdf]

Supporting Information

Strain-insensitive Viscoelastic Perovskite Film for Intrinsically Stretchable Neuromorphic Vision-Adaptive Transistors

Chengyu Wang[1,2], Yangshuang Bian[1,2], Kai Liu[1,2], Mingcong Qin[1,2], Fan Zhang[1,2], Mingliang Zhu[1,2], Wenkang Shi[1,2], Mingchao Shao[1,2], Shengcong Shang[1,2], Jiaxin Hong[1,2], Zhiheng Zhu[1,2], Zhiyuan Zhao[1,2], Yunqi Liu[1,2] and Yunlong Guo[1,2]*

*Corresponding author. Email: guoyunlong@iccas.ac.cn (Y.G.)

Supplementary Methods

Surface energy measurements

The surface energy of PDMS substrates, PQD films and all the conjugated polymer films were calculated according to the Owens-Wendt method[1]:

$$\gamma_s = \gamma_s^p + \gamma_s^d \tag{1}$$

$$(1+\cos\theta_l)\gamma_l = 2\left(\sqrt{\gamma_l^d \gamma_s^d} + \sqrt{\gamma_l^p \gamma_s^p}\right) \tag{2}$$

where γ_s, γ_s^p and γ_s^d were the total surface energy, polar component and dispersive component of surface energy, respectively. The total surface energy (γ_l), dispersion component (γ_l^d) and polar component (γ_l^p) were 50.8 mJ m^{-2}, 50.42 mJ m^{-2} and 0.38 mJ m^{-2} for diiodomethane, and 72.8 mJ m^{-2}, 29.1 mJ m^{-2} and 43.7 mJ m^{-2} for water, respectively.

Defect state density measurements

The defect-tunability of the perovskite film was certified according to hole-only device measurements and calculated as followed:

$$V_{TFL} = \frac{eN_t L^2}{2\varepsilon\varepsilon_0} \tag{3}$$

where e was the elementary charge, L was the thickness of the semiconductor film, ε_0 was the vacuum dielectric constant, and ε was the relative dielectric constant[40].

Electronic properties measurements

The mobility (μ) and threshold voltage (V_{th}) were important parameters for evaluating the electronic performances of OFETs. These values could be extracted from the curve of the drain current (I_{DS}) versus the gate voltage (V_G). In the saturation regime, the drain current (I_{DS}) versus the gate voltage (V_G) according to the following equation[2]:

$$I_{DS} = \frac{W}{2L} C_i \mu_{sat} (V_G - V_{th})^2 \tag{4}$$

where W and L represented the channel length and width, respectively. C_i was the capacitance of the gate dielectric.

The mobility was calculated by the slop of $\sqrt{|I_{DS}|}$ versus V_G according to the following equation[3]:

$$\mu_{sat} = \left(\frac{\partial\sqrt{|I_{DS}|}}{\partial V_G}\right)^2 \frac{2L}{WC_i} \tag{5}$$

$$|V_G - V_{th}| < |V_{DS}| \tag{6}$$

The V_{th} of the device was obtained by extrapolating a plot of $\sqrt{|I_{DS}|}$ versus V_G to $I_{DS} = 0$.

Works of adhesion measurements

The works of adhesion between different hybrid photosensitive films and nanoconfined semiconductor films were calculated as followed[4]:

$$W_{S1/S2} = 2\left(\sqrt{\gamma_{S1}^d \gamma_{S2}^d} + \sqrt{\gamma_{S1}^p \gamma_{S2}^p}\right) \tag{7}$$

where W, γ^d and γ^p were the calculated works of adhesion, polar component and dispersive component of measured surface energy, respectively.

Photosensitivity calculation for adaptive behaviors

To quantificationally evaluate the light response under different bias voltages (V_G) and light intensities (P_{in}), the photosensitivity (S_{ph}) was defined as follows:

$$S_{ph}=\frac{I_{ph}}{I_{dark}}=\frac{I_{illumination}\text{-}I_{dark}}{I_{dark}} \tag{8}$$

where I_{ph} was the difference between photocurrent and dark current. I_{dark} and $I_{illumination}$ represented the dark- and photo-current, respectively.

Fitting curves of the dependence between PPF and Δ*t*

The dependence of the extracted PPF index on the Δt could be well fitted by a double exponential decay function as follow[5]:

$$\text{PPF}=A_0+A_1\times\exp\left({}^{-\Delta t}/_{\tau_1}\right)+A_2\times\exp\left({}^{-\Delta t}/_{\tau_2}\right) \tag{9}$$

where A_1 and A_2 were rapid and slow facilitation magnitudes; τ_1 and τ_2 were rapid and slow decay time in PPF. In detail, A_1, A_2, τ_1 and τ_2 were calculated to be 3.14, 0.61, 0.50, and 19.08, respectively.

Fitting curves of the dependence between τ and *t*

The index (τ) referred to the relationship between the ratio of rise time to fall time, and it could be defined as follows:

$$\tau=\frac{t_{fall}}{t_{rise}} \tag{10}$$

where t_{rise} was the time for the current to rise from the dark state to the 90% of the maximum photocurrent, and t_{fall} was the time for the decay from the illumination just stopped to the 10% of its photocurrent.

The dependence between τ and lighting time (t) could also be well fitted by a double exponential decay function as follows:

$$\tau=A_0+A_1\times\exp\left({}^{-t}/_{\tau_1}\right)+\mathrm{A}_2\times\exp\left({}^{-t}/_{\tau_2}\right) \tag{11}$$

where A_1 and A_2 were rapid and slow facilitation magnitudes; τ_1 and τ_2 were rapid and slow decay time in τ. In detail, A_1, A_2, τ_1 and τ_2 were calculated to be 25.49, 1.78, 1.85, and 9.24, respectively.

Consumption calculation of synaptic behavior

The energy consumption (E) of typical synaptic behaviors could be calculated as follows[6]:

$$E=I_{peak}\times t\times V \tag{12}$$

where I_{peak}, t, and V were the peak value of EPSC, light pulse width, and operating voltage, respectively.

Supplementary Figures

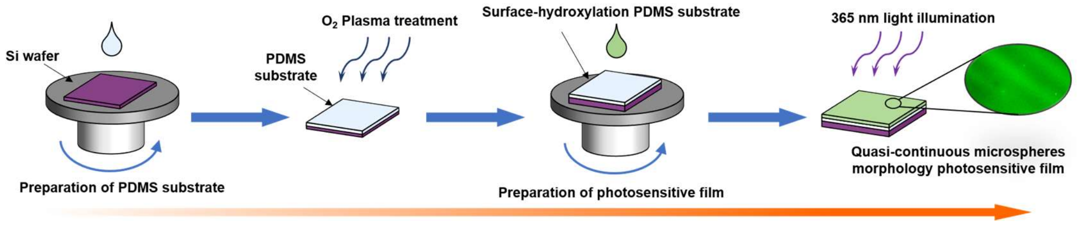

Supplementary Figure 1. Schematic illustration of the fabrication process for optimized photosensitive films with the QCM morphology.

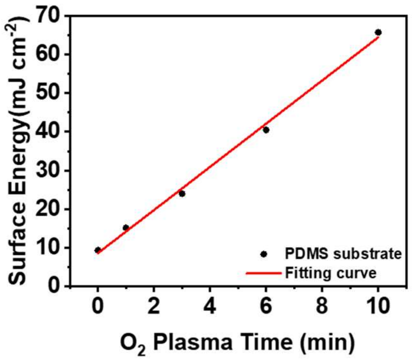

Supplementary Figure 2. Relationship between surface energy and O_2 plasma treatment time of PDMS substrates.

Supplementary Table 1. Surface energy evolution of the PDMS substrates under different treatment times of O_2 plasma.

Materials	O_2 plasma treatment	Contact angles (°)						Surface Energy (mJ m^{-2})		
		H_2O			CH_2I_2			γ_s	γ_s^d	γ_s^p
**PDMS Substrate**	**0 min**	112.5	114.35	113	99.85	99.05	98.9	**9.42**	8.52	0.9
	**1 min**	104.5	104.55	104.05	85.75	86.25	85.6	**15.17**	13.99	1.18
	**3 min**	93.9	94.65	92.55	69.5	68.35	70.05	**24.03**	22.37	1.67
	**6 min**	79.15	79.15	77.9	40.2	40	40.55	**40.49**	38.11	2.39
	**10 min**	29.1	28.8	29.7	32.6	33.2	33.3	**65.72**	37.38	28.34

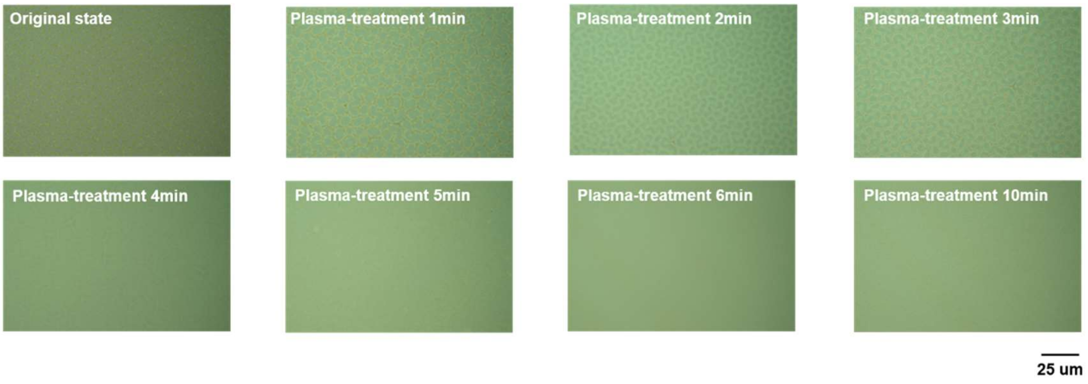

Supplementary Figure 3. Optical microscopes of the morphology evolution of the hybrid photosensitive films. The hybrid photosensitive solution (PQDs: SEBS=10:1) was spin-coated onto the PDMS substrates with the O_2 plasma treatment of 0 min, 1 min, 2 min, 3 min, 4 min, 6 min, 6 min and 10 min, respectively.

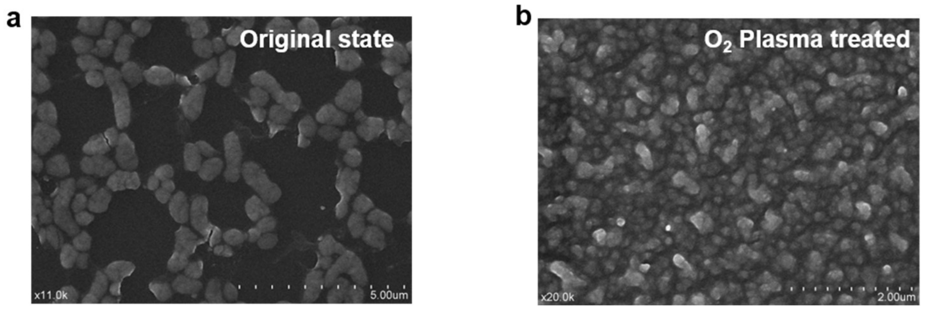

Supplementary Figure 4. Scanning electron microscope images of hybrid photosensitive films at different morphologies. **a** Original state with a spindle-like morphology. **b** O_2 plasma treated state with the QCM morphology. There was an obvious transformation from discontinuous morphologies to quasi-continuous morphologies with smaller PQD microspheres.

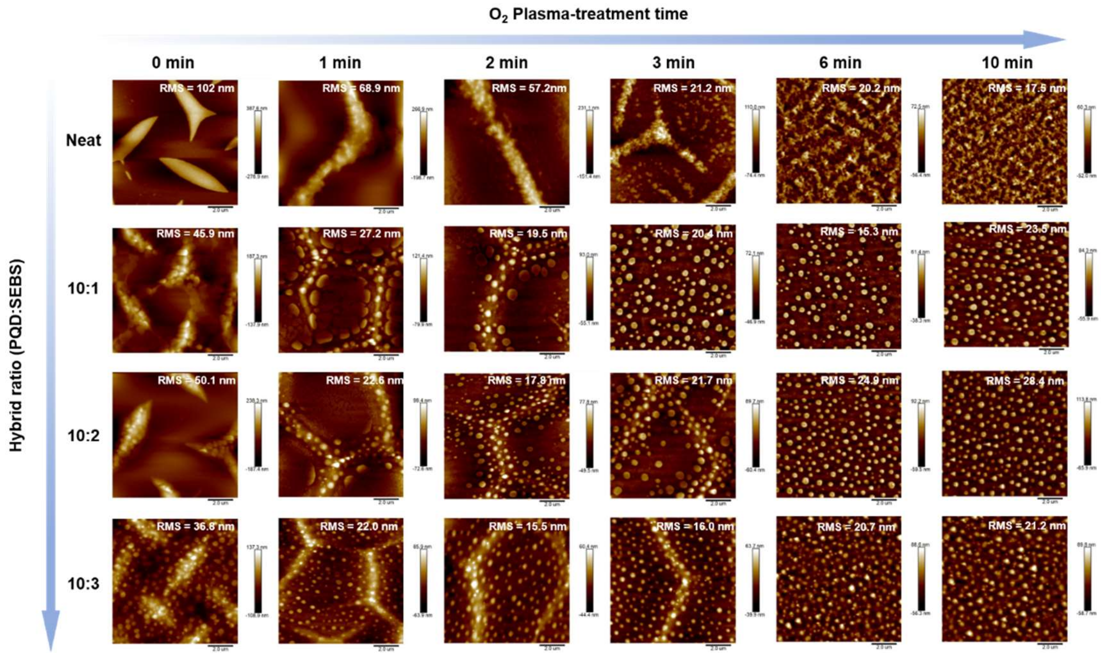

Supplementary Figure 5. Atomic force microscope (AFM) images of the hybrid photosensitive film morphologies with different hybrid ratios and O_2 plasma treatment times. The transforming states showed that the introduction of elastomer could wrap the perovskite quantum dots and greatly reduce the surface roughness. Moreover, as the O_2 plasma treatment time increased, the film morphologies could undergo an evolution process from a spindle-like morphology, then a honeycomb-like morphology, and eventually to a QCM morphology with gradually decreased surface roughness.

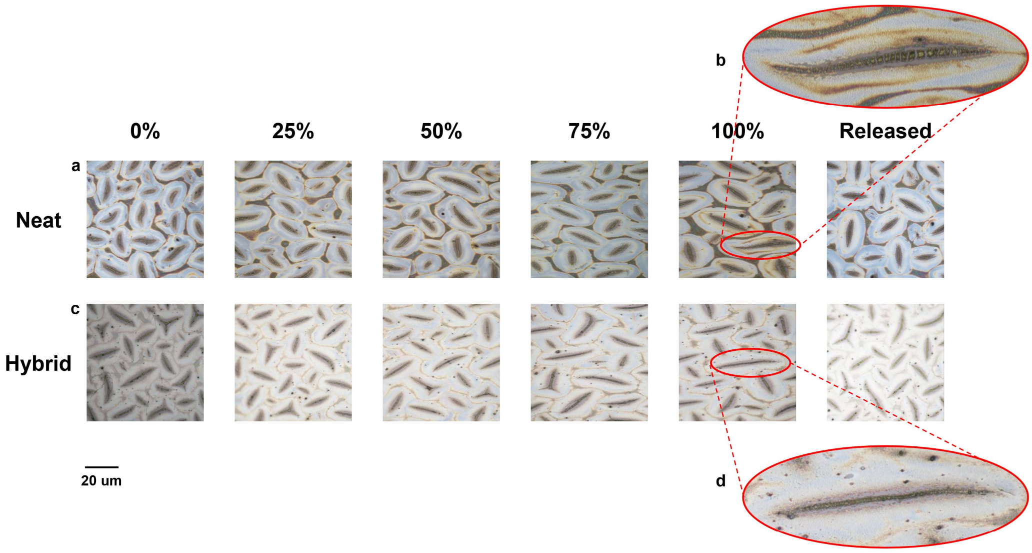

Supplementary Figure 6. Optical microscope images of the comparison between the neat PQD film and the PQD/SEBS hybrid film under different strain. **a** The neat film under various strain from 0% to 100%; **b** The enlarged detail of neat film under 100% strain; **c** the neat hybrid film under various strain from 0% to 100%; **d** The enlarged detail of hybrid film under 100% strain. It demonstrated obvious cracks in stretched neat film while the hybrid film did not, and the SEBS increased the viscoelasticity and stretchability.

Materials	Neat			Hybrid		
Treatment	0 min	3 min	6 min	0 min	3 min	6 min
Hight	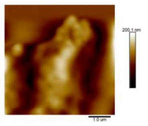	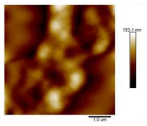	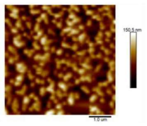	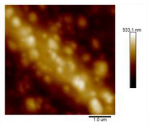	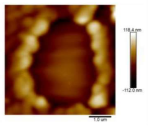	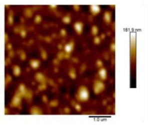
DMT Modulus	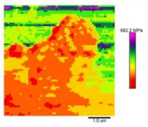	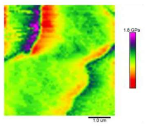	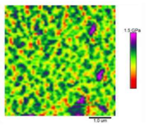	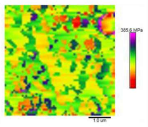	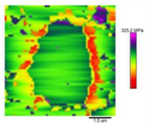	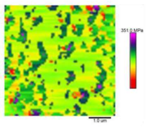
Modulus (MPa)	267	686	1244	69.7	191	113

Supplementary Figure 7. DMT modulus measurement of neat and hybrid perovskite films (PQDs : SEBS = 10:1, w/w) under different O_2 plasma treatment.

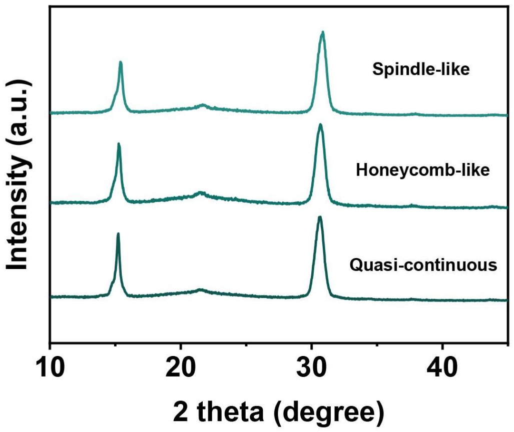

Supplementary Figure 8. X-ray diffraction diagrams of hybrid photosensitive films with different morphologies.

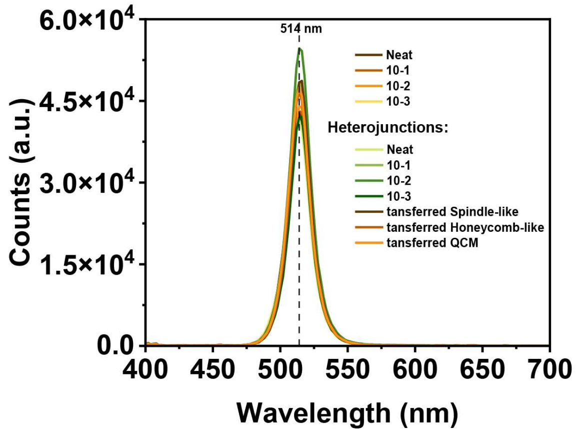

Supplementary Figure 9. Steady state photoluminescence (PL) spectra of photosensitive films with different O_2 plasma treatments and their corresponding layer heterojunctions.

Supplementary Table 2. Surface energy and works of adhesion between nanoconfined semiconductor layer and photosensitive films under different SEBS contents and O_2 plasma treatment times. (The introduced SEBS and a proper O_2 plasma treatment would together provide closer surface energy for the ideal interfacial contact between photosensitive films and semiconductor films, and better photogenerated charge transport and stretchability.)

Materials	O_2 plasma treatment	Surface Energy (mJ m^{-2})			Works of Adhesion(mJ m^{-2})
		γ_s^d	γ_s^p	γ_s	(with semiconductor layer)
Neat PQDs	6 min	26.69	19.07	**45.76**	60.12883638
PQDs:SEBS=10:1	0 min	13.94	2.64	**16.57**	42.58886004
	3 min	23.93	0.7	**24.63**	55.06926426
	6 min	29.76	0	**29.76**	60.88436252
PQDs:SEBS=10:2	6min	36.16	0.5	**36.66**	67.51251448
PQDs:SEBS=10:3	6min	36.57	0.77	**37.34**	67.98830646
DPPT-TT:SEBS=5:5		31.14	0.08	**31.22**	

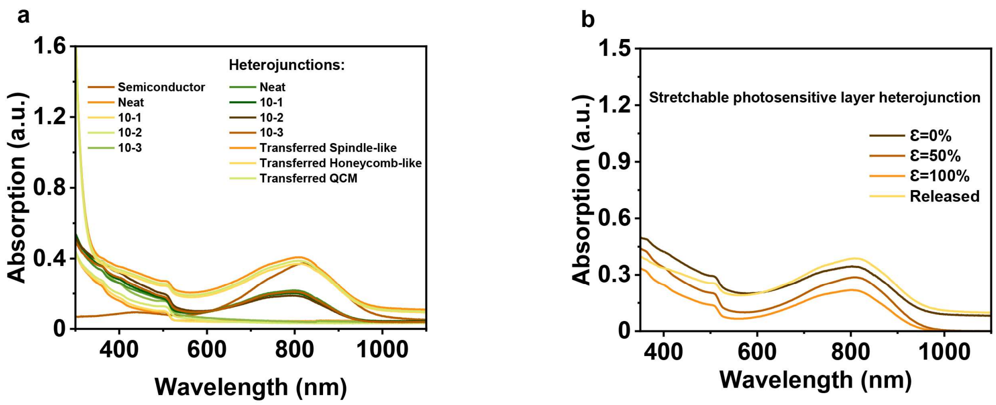

Supplementary Figure 10. a UV-vis absorption spectra of photosensitive films and layer heterojunctions with different hybrid ratios and different O_2 plasma treatments. **b** The photosensitive layer heterojunction under 0%, 50%, 100% strains and after the release.

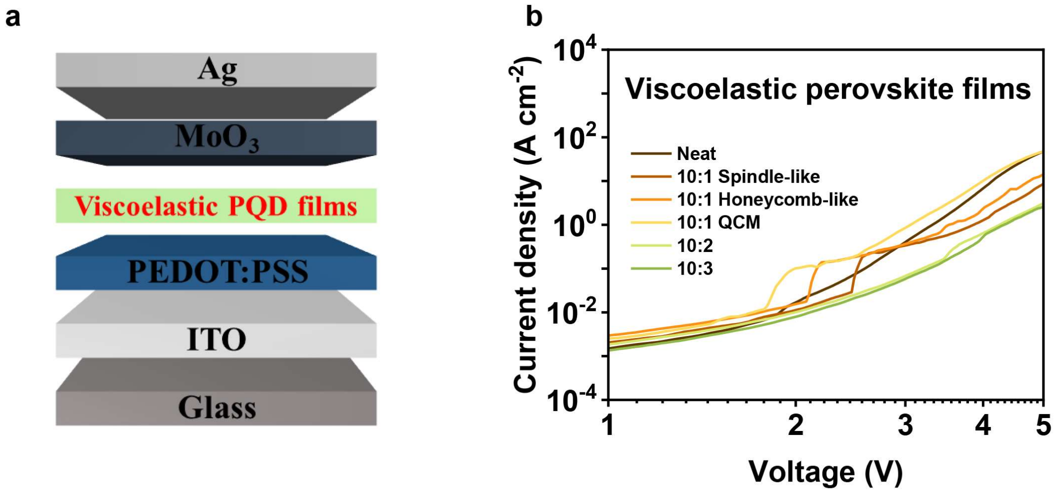

Supplementary Figure 11. Defect-tunability of different viscoelastic perovskite films. **a** Schematic diagram of device structure for the hole-only devices measurement; **b** SCLC curves of different viscoelastic perovskite films.

Supplementary Table 3. The V_{TFL} and N_t among various viscoelastic perovskite films.

	V_{TFL} (V)	N_t (cm^{-3})
Neat	1.71	5.62×10^{18}
10:1 Spindle-like	2.42	7.96×10^{18}
10:1 Honeycomb-like	2.07	6.81×10^{18}
10:1 QCM	1.76	5.79×10^{18}
10:2	3.03	9.96×10^{18}
10:3	3.28	1.08×10^{19}

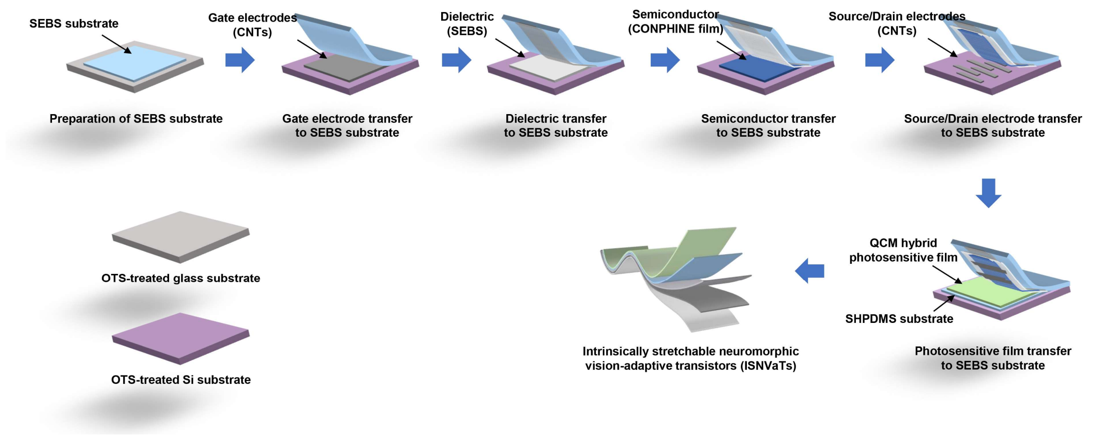

Supplementary Figure 12. Schematic illustration of the fabrication process for intrinsically stretchable neuromorphic vision-adaptive transistors (ISNVaTs).

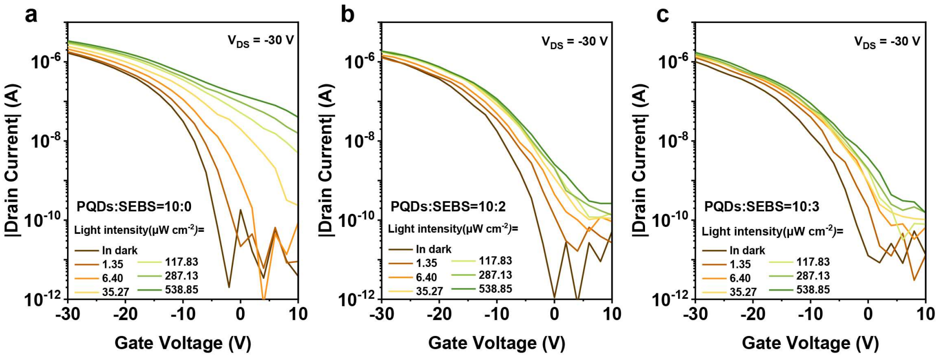

Supplementary Figure 13. Typical transfer curves of ISNVaTs under various light intensities with photosensitive films of different hybrid ratios (PQDs:SEBS). **a** The neat film. **b** Hybrid ratio of 10:2. **c** Hybrid ratio of 10:3. We selected the hybrid photosensitive films with similar quasi-continuous morphologies and surface roughness to avoid other variables.

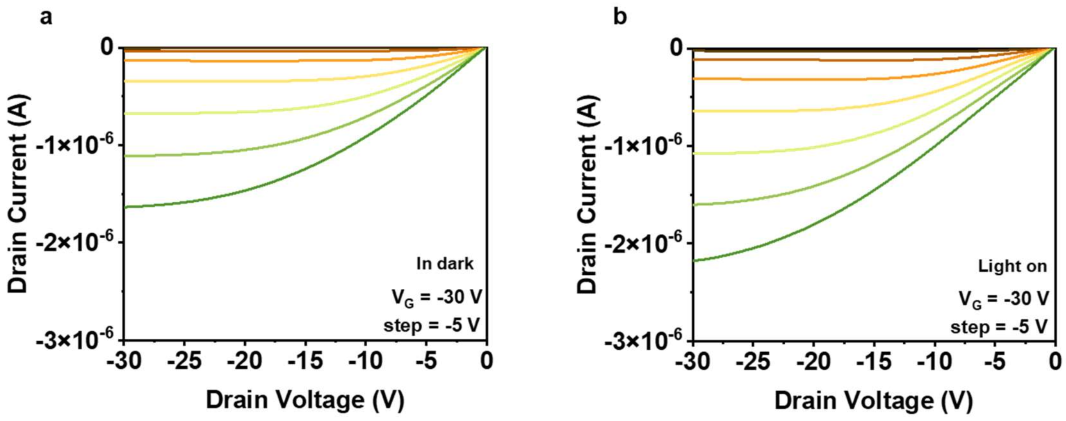

Supplementary Figure 14. Typical output curves of ISNVaTs. **a** In the dark state. **b** Under 365 nm light illumination at a P_{in} of 35.27 μW cm^{-2}.

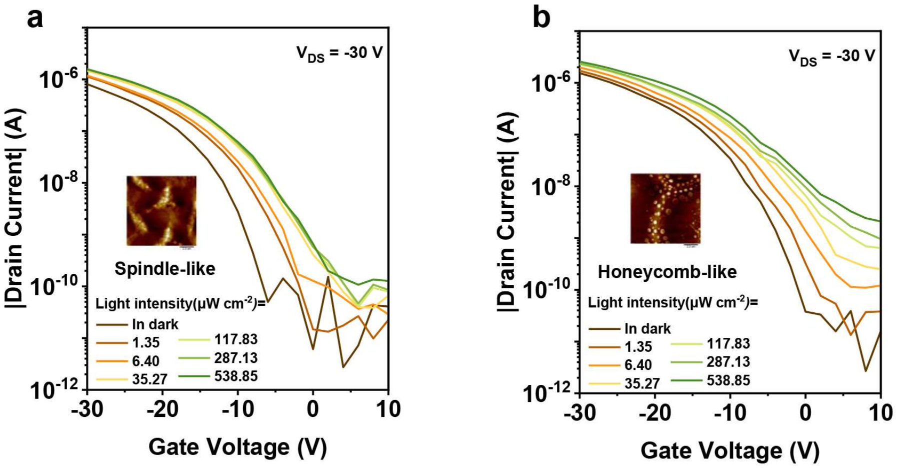

Supplementary Figure 15. Typical transfer curves of ISNVaTs under various light intensities with different morphologies at 10:1 hybrid ratio. **a** Typical transfer curves with spindle-like morphology photosensitive films. **b** Typical transfer curves with honeycomb-like morphology photosensitive films. The device with spindle-like morphology photosensitive film showed rather poor photoresponse because its high surface roughness severely hindered the photogenerated carrier transport. Similarly, the device with honeycomb-like morphology photosensitive film performed improved photoresponse and the QCM hybrid photosensitive film showed the best performance.

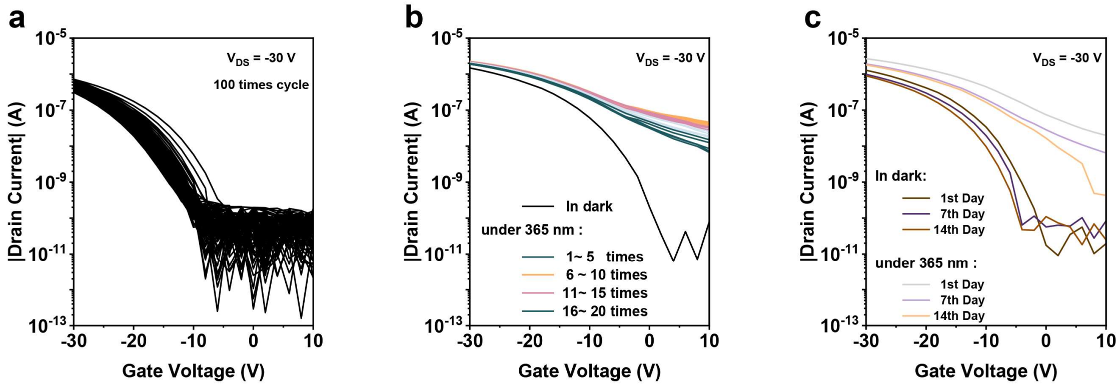

Supplementary Figure 16. Stability test of ISNVaTs. **a** Cycle test stability in the dark state; **b** Cycle test stability under 365 nm illumination (P_{in} = 538.85 μW cm^{-2}); **c** Temporal stability in nitrogen atmosphere for 14 days (P_{in} = 538.85 μW cm^{-2}).

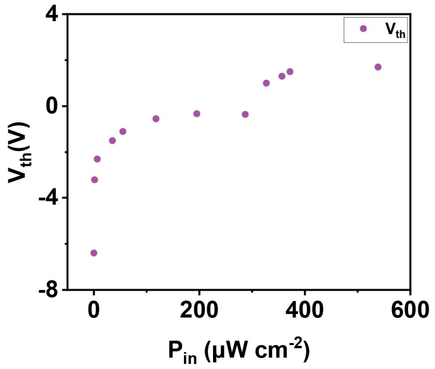

Supplementary Figure 17. The threshold voltage (V_{th}) under different light intensities (P_{in}). The increased light intensities favored the injection of photogenerated holes into the active layer channel, and the threshold voltage (V_{th}) of the devices drifted steadily from -6.4 to 1.7 V as the illumination was enhanced from 0 to 538 μW cm^{-2}, proving the stable light recognition.

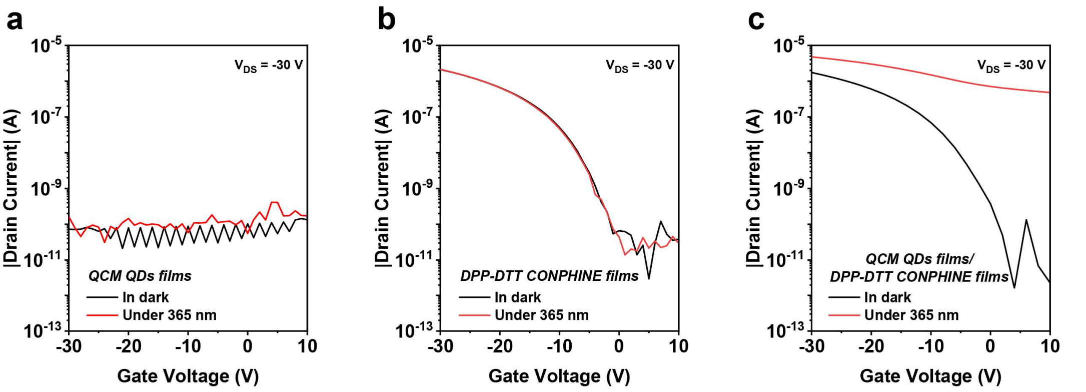

Supplementary Figure 18. Comparison of typical transfer curves on various active layers. **a** QCM QDs films; **b** DPP-DTT CONPHINE films; **c** QCM QDs films/DPP-DTT CONPHINE films.

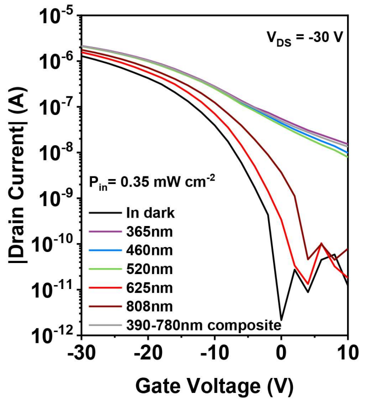

Supplementary Figure 19. Typical transfer curves of ISNVaTs under various light wavelengths at a P_{in} of 0.35 mW cm^{-2}.

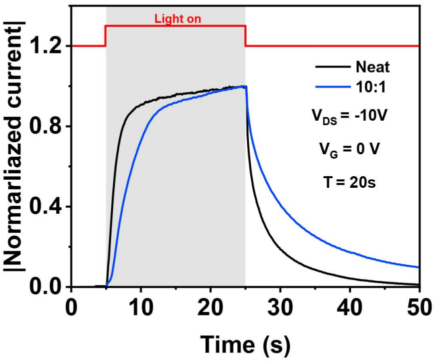

Supplementary Figure 20. Normalized *I-T* curves of the ISNVaTs based on neat PQD film and QCM hybrid photosensitive film at 10:1 hybrid ratio. As shown in the figure, the ISNVaT devices with elastic QCM hybrid photosensitive films displayed slower rise and recovery of photocurrent, indicating better synaptic and adaptive functions.

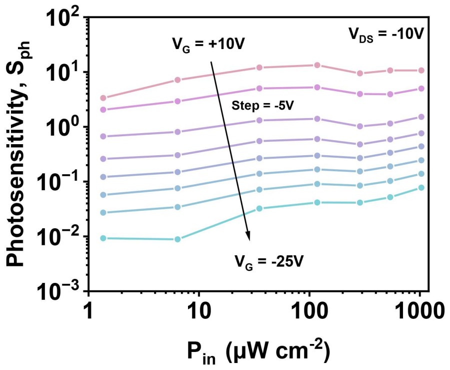

Supplementary Figure 21. Photosensitivity versus P_{in} under different V_G values. It demonstrated an obvious growth trend of S_{ph} with increasing P_{in} and higher values at positive gate bias. These results verified that light intensity and gate voltage could together modulate the photoresponse of the device, allowing the emulation of negative feedback functions in the visual system.

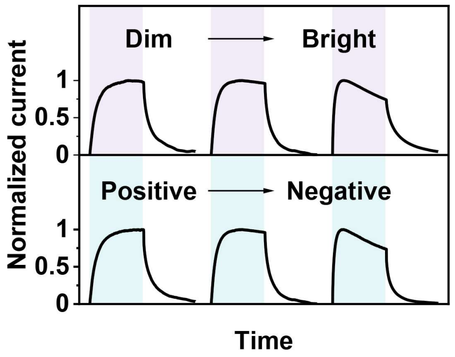

Supplementary Figure 22. Single-curve normalized current of light intensity dependence (top, V_G = 0) and gate voltage dependence (bottom, P_{in} = 356.69 μW cm^{-2}) adaptation behaviors of ISNVaTs. (dim: 6.41 μW cm^{-2}, normal: 356.69 μW cm^{-2}, bright: 698.09 μW cm^{-2}; positive: +10 V, normal: 0 V negative: -10 V)

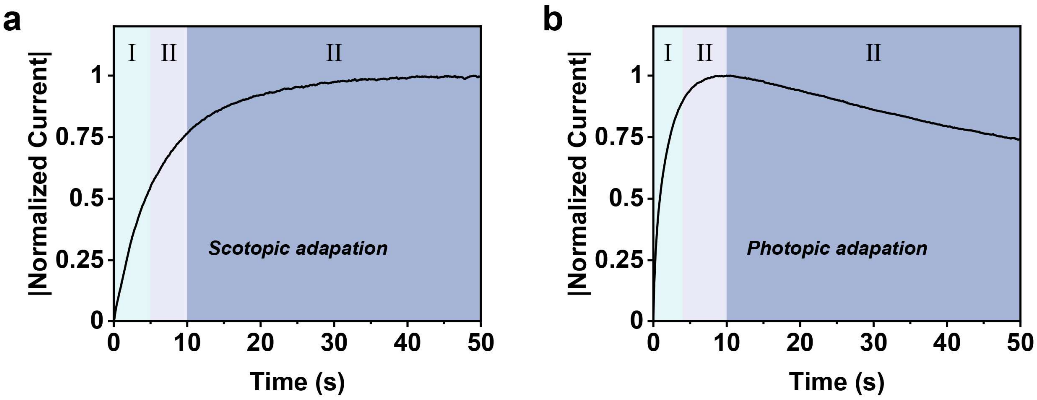

Supplementary Figure 23. Schematic diagram of the adaptation process. **a** Scotopic adaptation. **b** Photopic adaptation. (I. Reception and perception; II. Transformation; III. Adaptation.)

In the biological system, the transition point from cone-mediated to rod-mediated thresholds was termed as the rod-cone break, and it was almost about one fifth (10 min) of the entire adaptation time (45-50 min). Analogizing with biological adaptive systems, we categorized the adaptation into several processes: I. Reception and perception, II. Transformation, III. Adaptation. It could be noticed that the rod-cone break typically occurred near 10 s (the end of process II). Therefore, we defined CCR based on the photocurrent at 10 s rather than the initial current, which was believed to be more in consistent with the real situation time of visual adaptation.

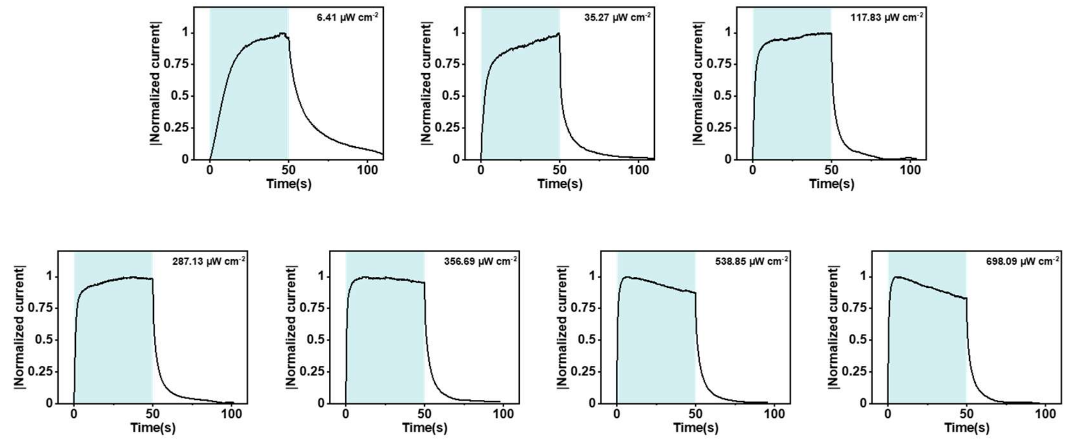

Supplementary Figure 24. Light-intensity-dependence adaptive behaviors. The ISNVaTs were applied 50 s-temporal illumination with different intensities (6.41, 35.27, 117.83, 287.13, 356.69, 538.85 and 689.09 μW cm^{-2}, respectively.) at a gate voltage of 0 V.

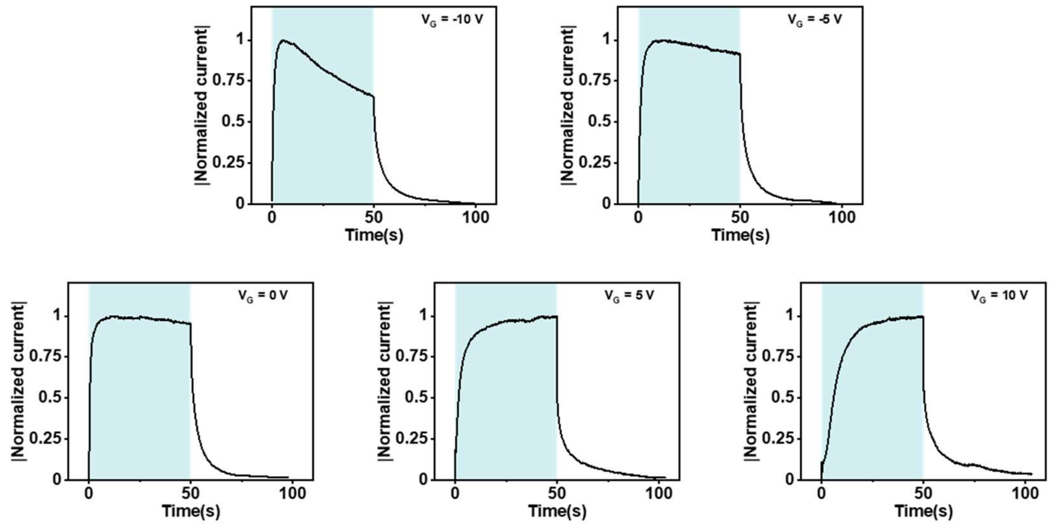

Supplementary Figure 25. Light-intensity-dependence adaptive behaviors. The ISNVaTs were applied 50 s-temporal illumination under 356.69 μW cm^{-2} at different gate voltages of -10 V, -5 V , 0 V , 5 V and 10 V, respectively. Notably, the illumination value of 356 μW cm^{-2} used in our manuscript can be converted to 427.2 lux, which is consistent with the comfortable indoor illumination on sunny days (100-1000 lux).

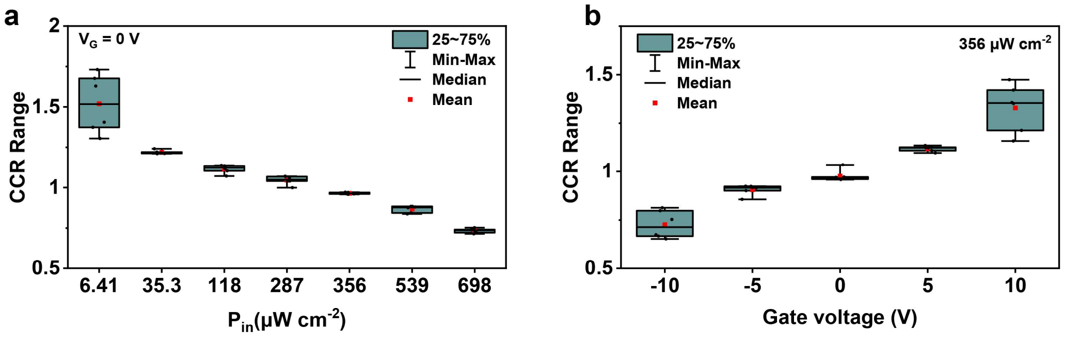

Supplementary Figure 26. Statistical data pots of adaptation behaviors. **a** Light-intensity dependence that CCR at different P_{in} values. **b** Gate-voltage dependence that CCR at different V_G values. (N=6)

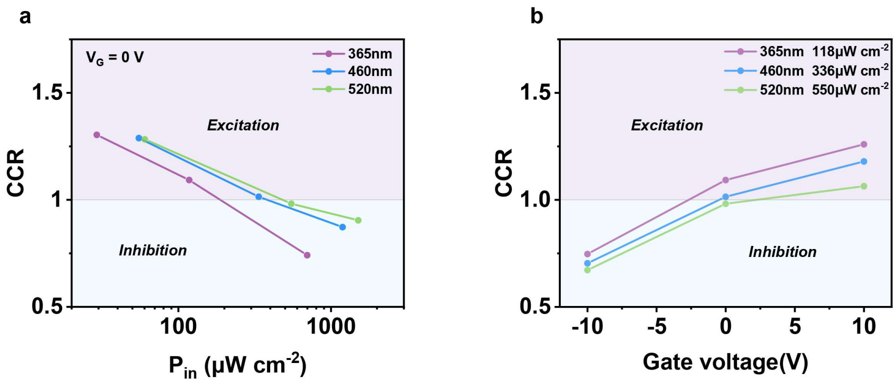

Supplementary Figure 27. a Extracted CCR at different P_{in} values. **b** Different V_G values under 365 nm, 460 nm and 520 nm light, respectively. The photoresponse of ISNVaT was lower under visible regions than ultraviolet light, resulting in weaker adaptive behaviors. Although with lower utilization of visible light, the ISNVaTs could still mimic the visual adaptation and be modulated by light intensity and gate voltage over multiple wavelengths.

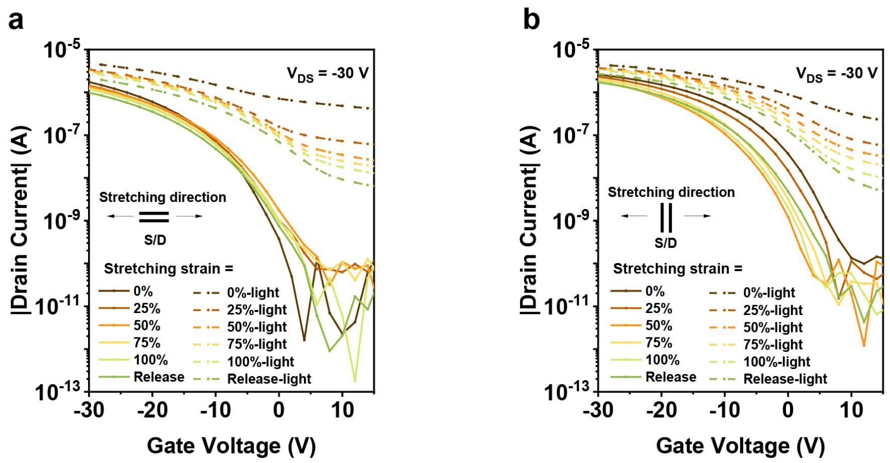

Supplementary Figure 28. Strain-tolerance characteristics of ISNVaTs. The ISNVaTs with QCM hybrid photosensitive films at 10:1 hybrid ratio were subjected to stretching strain from 0% to 100% in different direction. **a** Typical transfer curves under the stretching direction perpendicular to the charge transport direction. **b** Typical transfer curves under the stretching direction parallel to the charge transport direction.

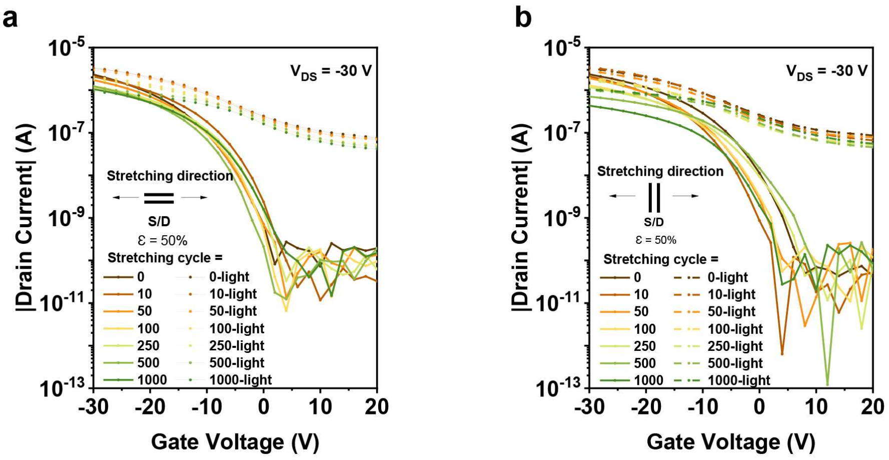

Supplementary Figure 29. Electrical stability characteristics against stretching cycle of ISNVaTs. The ISNVaTs with QCM hybrid photosensitive films at 10:1 hybrid ratio were subjected to multiple stretching-releasing cycles up to 1000 times at 50% strain in different direction. **a** Typical transfer curves under the stretching direction perpendicular to the charge transport direction. **b** Typical transfer curves under the stretching direction parallel to the charge transport direction.

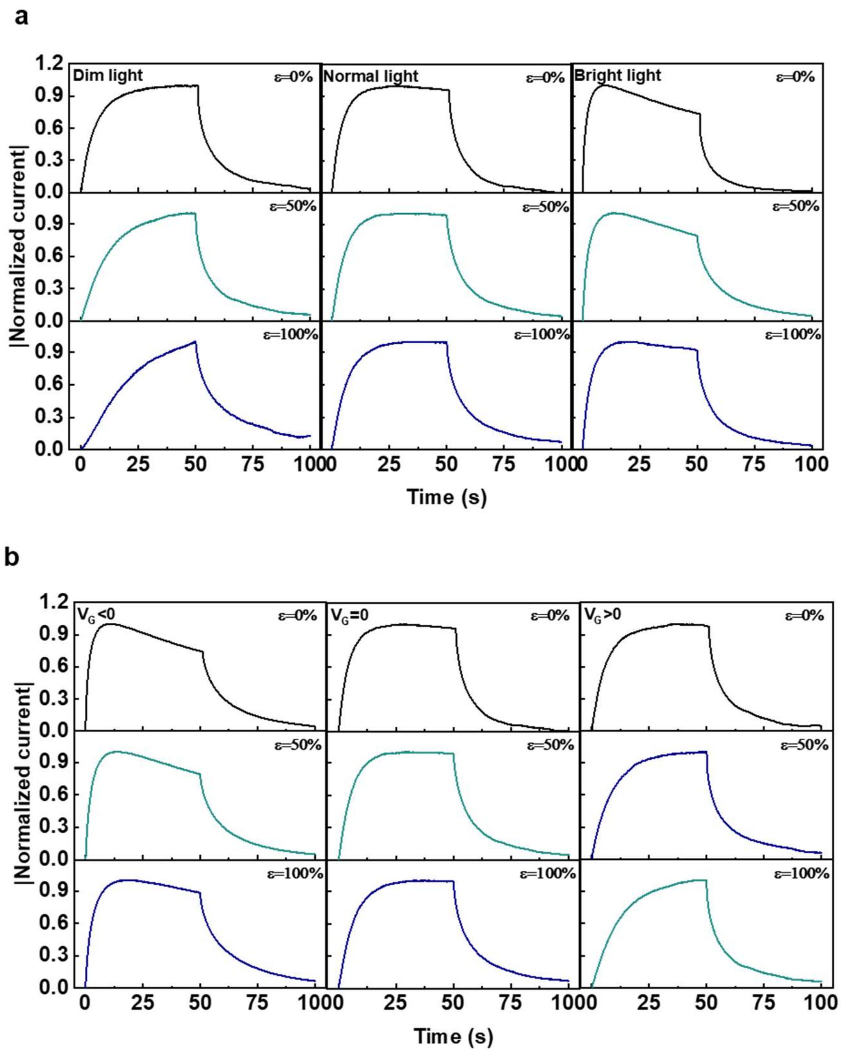

Supplementary Figure 30. Adaptive behaviors of ISNVaTs under different strains. A 50-second temporal response was provided to evaluate the dependence of adaptive behaviors of ISNVaTs under the 0%, 50% and 100% stretching strain, respectively. **a** Light-intensity-dependence. **b** Gate-voltage-dependence.

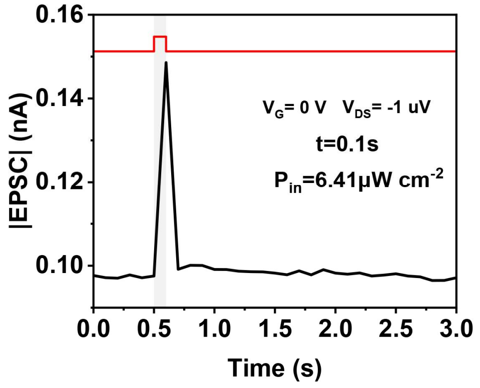

Supplementary Figure 31. EPSC triggered by the single light pulse of the ISNVaT at V_{DS} = -1 μV. The energy consumption was calculated from a typical synaptic behavior and an ultra-low power consumption of 15 aJ was obtained.

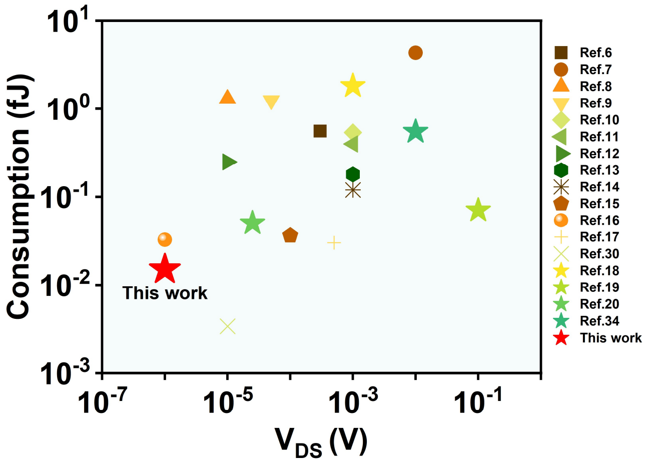

Supplementary Figure 32. Consumption comparison of previously reported organic synaptic phototransistors with our ISNVaT device.

Supplementary Table 4. List of previously reported organic synaptic phototransistors with our ISNVaT device.

Active layer	Stretchability	Consumption (fJ)	V_{DS} (V)	Duration (s)	Reference
HTEB	Rigid	4.29	-1×10^{-2}	0.05	[7]
$CsPbBr_3$/PDPP4T	Rigid	1.3	-1×10^{-5}	0.5	[8]
Pentacene/PVSK-PhB	Rigid	1.25	-5×10^{-5}	1	[9]
P3HT-b-P2VP	Rigid	0.56	-3×10^{-4}	0.2	[6]
DNTT	Rigid	0.54	-1×10^{-3}	0.05	[10]
DNTT/MoS_2	Rigid	0.4	-1×10^{-3}	0.02	[11]
Chlorophyll/PDPP4T	Rigid	0.25	-1×10^{-5}	0.05	[12]
$CsPbBr_3$ QDs/P3HT/CNFs	Rigid	0.18	-1×10^{-3}	0.05	[13]
DPPDTT/SEBS	Rigid	0.12	-1×10^{-3}	N/A	[14]
$CsPbBr_3$ QDs /TIPS	Rigid	0.036	-1×10^{-4}	0.05	[15]
PV2T-BT	Rigid	0.0324	-1×10^{-6}	0.2	[16]
P3HT/$FAPbBr_3$ QD	Rigid	0.03	-5×10^{-4}	0.05	[17]
PDP4T/carotene	Rigid	0.0034	-1×10^{-5}	0.01	[30]
P3HT-b-PPI(5F)/PMMA	Flexible	1.82	-1×10^{-3}	1	[18]
PbS QDs/PMMA/Pentacene	Flexible	0.55	-1×10^{-2}	0.1	[34]
Dif-TES-ADT	Flexible	0.07	-1×10^{-1}	0.25	[19]
C8-BTBT/P(VDT-TrFE)	Flexible	0.05	-2.5×10^{-5}	0.02	[20]
$CsPbBr_3$ QCM film /DPP-DTT CONPHINE film	100% Stretchable	0.015	-1×10^{-6}	0.1	This work

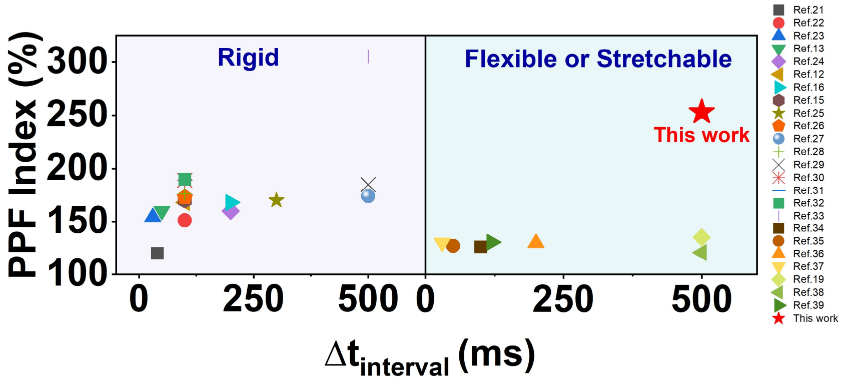

Supplementary Figure 33. Comparison of the PPF index among reported synaptic phototransistors prepared by different organic semiconductors.

Supplementary Table 5. Comparison of the PPF index among synaptic phototransistors prepared by different organic semiconductors.

Active layer	Stretchability	PPF index(%)	Δt (ms)	Reference
NT-CN/PMMA/Pentacene	Rigid	120	40	[21]
$CsPbBr_3$ QDs/PVP/PDVT-10	Rigid	151	100	[22]
Si NCs/P3HT	Rigid	154	30	[23]
$CsPbBr_3$ QDs/P3HT/CNFs	Rigid	160	50	[13]
$CsPbBr_3$ QDs/TIPS	Rigid	160	200	[24]
Chlorophyll/PDPP4T	Rigid	168*	100*	[12]
PV2T-BT	Rigid	168	200	[16]
$CsPbBr_3$ QDs /TIPS	Rigid	170	100	[15]
$CsPbBr_3$ QDs/DPPDTT	Rigid	170	300	[25]
$CsPbBr_3$ /P3HT	Rigid	173*	100*	[26]
$CsPbBr_3$ QDs/PDVT-10	Rigid	174	500	[27]
PAN/C8-BTBT	Rigid	176	100	[28]
P3HT/PCBM/Ion gels	Rigid	185	500	[29]
PDP4T/carotene	Rigid	189	100	[30]
PDPP3T/Chitosan	Rigid	190	100	[31]
P3HT	Rigid	190*	100*	[32]
C_{12}-BTBT/P-FPS	Rigid	305*	500	[33]
PbS QDs/PMMA/Pentacene	Flexible	129*	100*	[34]
PbS QDs/PDPP-C_6Si	Flexible	130*	50*	[35]
P3HT-b-MH/BCHL	Flexible	133	200	[36]
DNTT/OPP	Flexible	133.5	30	[37]
Dif-TES-ADT	Flexible	139	500	[19]
IDT-BT	100% Stretchable	123	500	[38]
P3HT NWs/Ion gels	100% Stretchable	134	120	[39]
$CsPbBr_3$ QCM film /DPP-DTT CONPHINE film	100% Stretchable	270	600	This work

The "*" in table indicated that the PPF indexes were not published directly and were derived by the given equations and graphs.

Supplementary Table 6. Comparison of the PPF index and consumption among synaptic phototransistors prepared by different organic semiconductors.

Active layer	Stretchability	Consumption (fJ)	$\|V_{DS}\|$ (V)	PPF index(%)	Δt (ms)	Reference
P3HT-b-P2VP	Rigid	0.56	3×10^{-4}	138	100	[6]
HTEB	Rigid	4.29	1×10^{-2}	210*	10	[7]
$CsPbBr_3$/PDPP4T	Rigid	1.3	1×10^{-5}	146.5*	100	[8]
Pentacene/PVSK-PhB	Rigid	1.25	5×10^{-5}	176*	100	[9]
DNTT	Rigid	0.54	1×10^{-3}	206	50	[10]
DNTT/MoS_2	Rigid	0.4	1×10^{-3}	113.5*	500	[11]
Chlorophyll/PDPP4T	Rigid	0.25	1×10^{-5}	168*	50	[12]
$CsPbBr_3$ QDs/P3HT/CNFs	Rigid	0.18	1×10^{-3}	160	50	[13]
DPPDTT/SEBS	Rigid	0.12	1×10^{-3}	124*	400*	[14]
$CsPbBr_3$ QDs /TIPS	Rigid	0.036	1×10^{-4}	170*	100*	[15]
PV2T-BT	Rigid	0.0324	1×10^{-6}	168	200	[16]
P3HT/$FAPbBr_3$ QD	Rigid	0.03	5×10^{-4}	196*	100*	[17]
NT-CN/PMMA/Pentacene	Rigid	18.06	3×10^{-1}	120	40	[21]
$CsPbBr_3$ QDs/TIPS	Rigid	76	1×10^{-3}	160	200	[24]
$CsPbBr_3$ QDs/DPPDTT	Rigid	0.5	5×10^{-4}	170	300	[25]
PDP4T/carotene	Rigid	0.0034	1×10^{-5}	189	100	[30]
P3HT-b-PPI(5F)/PMMA	Flexible	1.82	1×10^{-3}	118*	1000	[18]
Dif-TES-ADT	Flexible	0.07	1×10^{-1}	139	500	[19]
C8-BTBT/P(VDT-TrFE)	Flexible	0.05	2.5×10^{-5}	121*	500	[20]
PbS QDs/PMMA/Pentacene	Flexible	0.55	1×10^{-2}	129*	100*	[34]
$CsPbBr_3$ QCM film /DPP-DTT CONPHINE film	100% Stretchable	0.015	1×10^{-6}	270	500	This work

The "*" in table indicated that the PPF indexes were not published directly and were derived by the given equations and graphs.

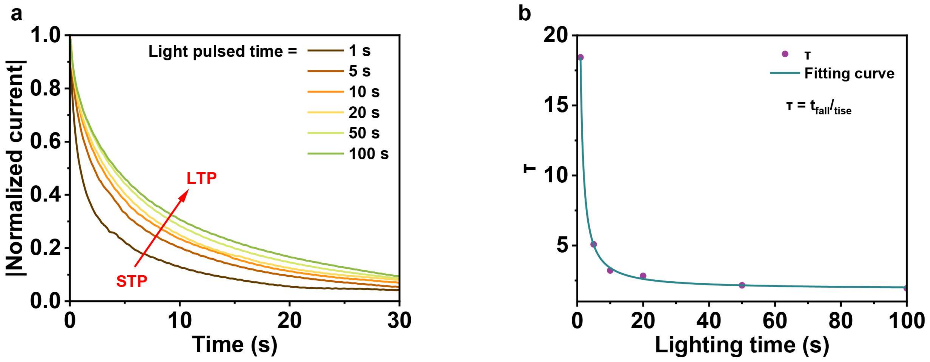

Supplementary Figure 34. Synaptic behaviors of the ISNVaTs. **a** Transformation from STP to LTP by modulating the light pulse duration. **b** Extracted τ as a function of lighting time.

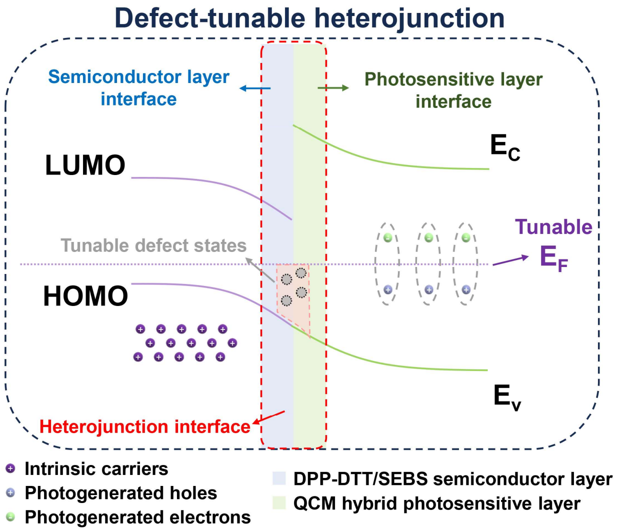

Supplementary Figure 35. Mechanism diagram of defect-tunable heterojunction.

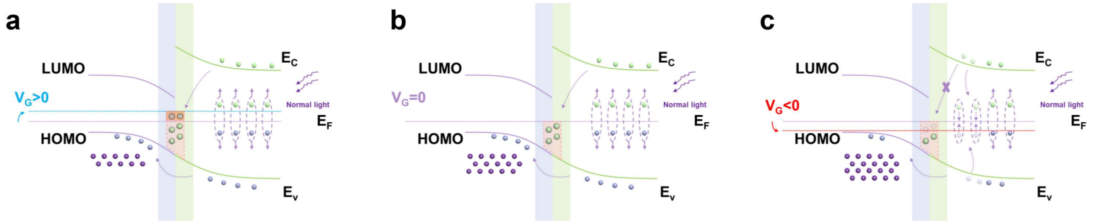

Supplementary Figure 36. Mechanism diagram of gate-voltage dependence. **a** Adaptation under positive gate voltage; **b** Adaptation under zero gate voltage; **c** Adaptation under negative gate voltage.

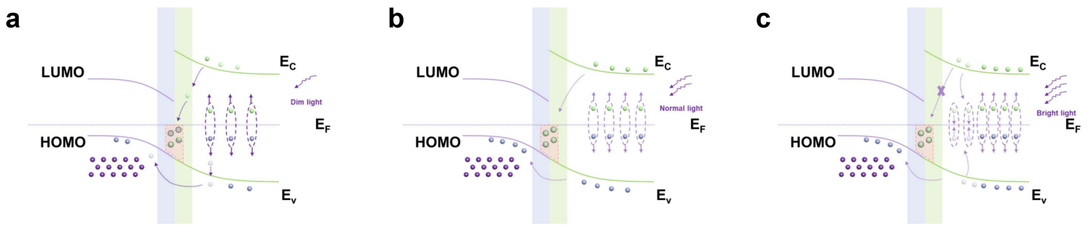

Supplementary Figure 37. Mechanism diagram of light-intensity dependence. **a** Adaptation under dim light; **b** Adaptation under normal light; **c** Adaptation under bright light.

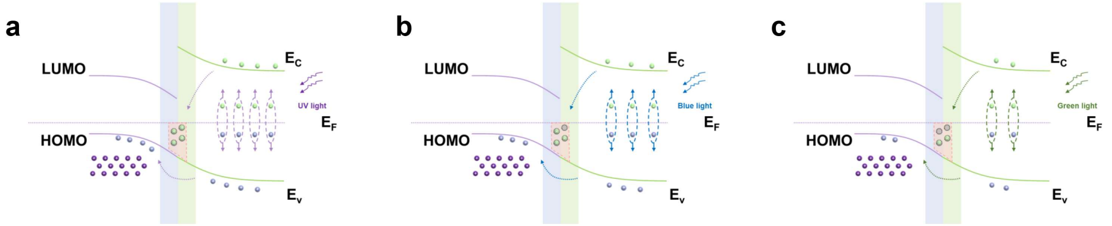

Supplementary Figure 38. Mechanism diagram of wavelength dependence. **a** Adaptation under UV light; **b** Adaptation under blue light; **c** Adaptation under green light.

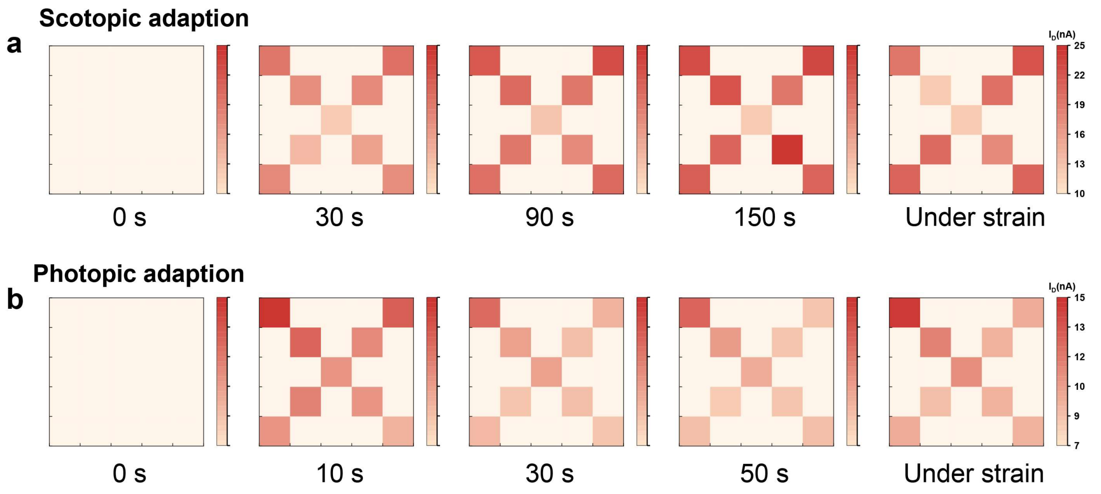

Supplementary Figure 39. Visual adaptation imagination of the 5*5 ISNVaT array under different gate voltage. **a** Scotopic adaptation (V_G = +10 V, P_{in} = 356.69 μW cm^{-2}); **b** Photopic adaptation (V_G = -10 V, P_{in} = 356.69 μW cm^{-2}). (The device at the center was applied at V_G = -10 V as a comparison.)

Supplementary Table 7. Comparison of adaptive time among recent related works.

Active layer	Stretchability	Semiconductor type	Visual adaptation	Adaptation time (s)	Reference
$CsPbBr_3$ QDs /MoS_2	Rigid	2D	Photopic adaptation	> 4	[41]
CsFAMA	Rigid	2D	Photopic adaptation	4.8	[42]
$CsPb(Br_{1-x}I_x)_3/MoS_2$	Rigid	2D	Photopic adaptation	> 60	[43]
MOF	Rigid	2D	Photopic adaptation<br>Scotopic adaptation	10<br>10	[44]
MoS_2	Rigid	2D	Photopic adaptation<br>Scotopic adaptation	80<br>10	[45]
Graphene/PbS QDs/graphene	Rigid	2D	Photopic adaptation<br>Scotopic adaptation	100s	[46]
CdSe&IGZO	Rigid	Inorganic oxide	Photopic adaptation<br>Scotopic adaptation	10<br>10	[47]
$MAPbI_3$&IZO	Rigid	Inorganic oxide	Photopic adaptation<br>Scotopic adaptation	> 40	[48]
MoO_3/LiTFSI	Rigid	Inorganic oxide	Photopic adaptation<br>Scotopic adaptation	> 50	[49]
InP QDs/ITZO	Rigid	Inorganic oxide	Photopic adaptation<br>Scotopic adaptation	300<br>300	[50]
Two bulk heterojunctions	Rigid	Organic	Photopic adaptation	2	[51]
$CsPbBr_xI_{3-x}$/TIPS	Rigid	Organic	Photopic adaptation<br>Scotopic adaptation	> 300	[52]
$CsPbBr_3$ QCM film<br>/DPP-DTT CONPHINE film	100% Stretchable	Organic	Photopic adaptation<br>Scotopic adaptation	< 150	This work

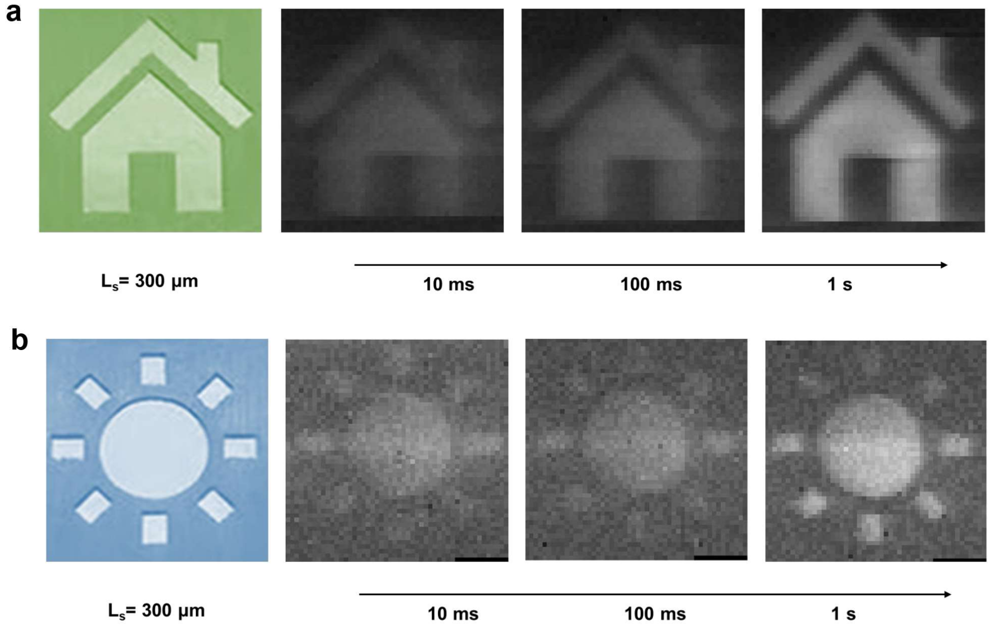

Supplementary Figure 40. Single-pixel adaptive imaging of ISNVaTs. **a** Simulated visual adaptation results under green light with a house-shaped mask; **b** Simulated visual adaptation results under blue light with a sun-shaped mask. (illumination intervals: 10 ms, 100 ms and 1 s)

Supplementary Reference

1. Xu, J. et al. Highly stretchable polymer semiconductor films through the nanoconfinement effect. *Science* **355**, 59-64 (2017).
2. Guo, Y.L. et al. Functional Organic Field-Effect Transistors. *Adv. Mater.* **22**, 4427-4447 (2010).
3. Xu, J. et al. Multi-scale ordering in highly stretchable polymer semiconducting films. *Nat. Mater.* **18**, 594-602 (2019).
4. Vohra, V. et al. Transfer-printing of active layers to achieve high quality interfaces in sequentially deposited multilayer inverted polymer solar cells fabricated in air. *Sci. Technol. Adv. Mater.* **17**, 530-540 (2016).
5. Han, C. et al. Light-Stimulated Synaptic Transistor with High PPF Feature for Artificial Visual Perception System Application. *Adv. Funct. Mater.* **32**, 2113053 (2022).
6. Yang, W.C. et al. Low-Energy-Consumption and Electret-Free Photosynaptic Transistor Utilizing Poly(3-hexylthiophene)-Based Conjugated Block Copolymers. *Adv.Sci.* **9**, 2105190 (2022).
7. Hua, Z. et al. Monolayer molecular crystals for low-energy consumption optical synaptic transistors. *Nano. Res.* **15**, 7639-7645 (2022).
8. Chen, T. et al. Photonic Synapses with Ultra-Low Energy Consumption Based on Vertical Organic Field-Effect Transistors. *Adv. Optical. Mater.* **9**, 2002030 (2021).
9. Wang, X. et al. Dye-sensitized perovskite/organic semiconductor ternary transistors for artificial synapses. *Sci. China. Mater.* **65**, 2521-2528 (2022).
10. Ercan, E. et al. Molecular template growth of organic heterojunctions to tailor visual neuroplasticity for high performance phototransistors with ultralow energy consumption. *Nanoscale. Horiz.* **8**, 632-640 (2023).
11. Wang, J. et al. Weak Light-Stimulated Synaptic Transistors Based on MoS_2/Organic Semiconductor Heterojunction for Neuromorphic Computing. *Adv. Mater. Technol.* **8**, 2300449 (2023).
12. Yang, B. et al. Bioinspired Multifunctional Organic Transistors Based on Natural Chlorophyll/Organic Semiconductors. *Adv. Mater.* **32**, 200122 (2020).
13. Ercan, E. et al. Self-Assembled Nanostructures of Quantum Dot/Conjugated Polymer Hybrids for Photonic Synaptic Transistors with Ultralow Energy Consumption and Zero-Gate Bias. *Adv. Funct. Mater.* **32**, 2107925 (2022).
14. Hao, D. et al. Artificial optoelectronic synaptic devices based on vertical organic field-effect transistors with low energy consumption. *Adv Compos Hybrid Mater* **6**, 129 (2023).
15. Shen, Z. et al. Ultralow-power consumption photonic synapse transistors based on organic array films fabricated using a particular prepatterned-guided crystallizing strategy. *J. Mater. Chem. C.* **11**, 3213-3226 (2023).
16. Yang, W.C. et al. High-Performance Organic Photosynaptic Transistors Using Donor-Acceptor Type and Crosslinked Core-Shell Nanoparticles as a Floating Gate Electret. *Adv. Optical Mater.* **11**, 2202110 (2023).
17. Chen, J.Y. et al. Ultrafast Responsive and Low-Energy-Consumption Poly(3-hexylthiophene)/Perovskite Quantum Dots Composite Film-Based Photonic Synapse. *Adv. Funct. Mater.* **31**, 2105911 (2021).
18. Jiang, L. et al. One-Step Preparation of Semiconductor/Dielectric Bilayer Structures for the Simulation of Flexible Bionic Photonic Synapses. *ACS. Appl. Mater. Interfaces.* **15**, 7227-7235

(2023).

19. Shi, J. et al. A Fully Solution-Printed Photosynaptic Transistor Array with Ultralow Energy Consumption for Artificial-Vision Neural Networks. *Adv. Mater.* **34**, 220038 (2022).
20. Li, Q. et al. Ultralow Power Wearable Organic Ferroelectric Device for Optoelectronic Neuromorphic Computing. *Nano Lett.* **22**, 6435-6443 (2022).
21. Park, H.L. et al. Retina-Inspired Carbon Nitride-Based Photonic Synapses for Selective Detection of UV Light. *Adv. Mater.* **32**, 1906899 (2020).
22. He, W. et al. A multi-input light-stimulated synaptic transistor for complex neuromorphic computing. *J. Mater. Chem. C.* **7**, 12523-12531 (2019).
23. Wang, Y. et al. Dual-Modal Optoelectronic Synaptic Devices with Versatile Synaptic Plasticity. *Adv. Funct. Mater.* **32**, 2107973 (2022).
24. Liu, J. et al. Weak Light-Stimulated Synaptic Hybrid Phototransistors Based on Islandlike Perovskite Films Prepared by Spin Coating. *ACS. Appl. Mater. Interfaces.* **13**, 13362-13371 (2021).
25. Hao, D. et al. Perovskite/Organic Semiconductor-Based Photonic Synaptic Transistor for Artificial Visual System. *ACS. Appl. Mater. Interfaces.* **12**, 39487-39495 (2020).
26. Gupta, G.K. et al. Inorganic Perovskite Quantum Dot-Mediated Photonic Multimodal Synapse. *ACS. Appl. Mater. Interfaces.* **15**, 18055-18064 (2023).
27. Wang, C. et al. $CsPbBr_3$ quantum dots/PDVT-10 conjugated polymer hybrid film-based photonic synaptic transistors toward high-efficiency neuromorphic computing. *Sci China Mater.* **65**, 3077-3086 (2022).
28. Dai, S. et al. Light-Stimulated Synaptic Devices Utilizing Interfacial Effect of Organic Field-Effect Transistors. *ACS. Appl. Mater. Interfaces.* **10**, 21472-21480 (2018).
29. Chen, K. et al. Organic optoelectronic synapse based on photon-modulated electrochemical doping. *Nat. Photon.* **17**, 629-637 (2023).
30. Yang, B. et al. Low-power consumption light-stimulated synaptic transistors based on natural carotene and organic semiconductors. *Chem. Commun.* **57**, 8300-8303 (2021).
31. Zang, Y. et al. A Dual-Organic-Transistor-Based Tactile-Perception System with Signal-Processing Functionality. *Adv. Mater.* **29**, 1606088 (2017).
32. Jiang, L. et al. Deep Ultraviolet Light Stimulated Synaptic Transistors Based on Poly(3-hexylthiophene) Ultrathin Films. *ACS. Appl. Mater. Interfaces.* **14**, 11718-11726 (2022).
33. Zhu, Y. et al. Side-Chain Engineering of Polystyrene Dielectrics Toward HighPerformance Photon Memories and Artificial Synapse. *Chem. Mater.* **34**, 6505-6517 (2022).
34. Zhang, J. et al. Retina-Inspired Artificial Synapses with Ultraviolet to Near-Infrared Broadband Responses for Energy-Efficient Neuromorphic Visual Systems. *Adv. Funct. Mater.* **8**, 2302885 (2023).
35. Huang, X. et al. Short-Wave Infrared Synaptic Phototransistor with Ambient Light Adaptability for Flexible Artificial Night Visual System. *Adv. Funct. Mater.* **33**, 2208836 (2023).
36. Ercan, E. et al. Harnessing Biobased Materials in Photosynaptic Transistors with Multibit Data Storage and Panchromatic Photoresponses Extended to Near-Infrared Band. *Adv. Optical. Mater.* **10**, 2201240 (2022).
37. Zhang, C. et al. Natural polyelectrolyte-based ultraflexible photoelectric synaptic transistors for hemispherical high-sensitive neuromorphic imaging system. *Nano Energy.* **95**, 107001

(2022).
38. Xu, F. et al. Intrinsically stretchable photonic synaptic transistors for retina-like visual image systems. *J. Mater. Chem. C.* **10**, 10586-10594 (2022).
39. Lee, Y. et al. Stretchable organic optoelectronic sensorimotor synapse. *Sci. Adv.* **4**, eaat7387 (2018).
40. Chen, K. et al. Solution-Processed $CsPbBr_3$ Quantum Dots/Organic Semiconductor Planar Heterojunctions for High-Performance Photodetectors. *Adv. Sci.* **9**, 2105856 (2022).
41. Xie, D. et al. Photoelectric visual adaptation based on 0D-$CsPbBr_3$-Quantum-Dots/2D-MoS_2 mixed-dimensional heterojunction transistor. *Adv. Funct. Mater.* **31**, 201065 (2021).
42. Chen, Q. et al. Switchable perovskite photovoltaic sensors for bioinspired adaptive machine vision. *Adv. Intell. Syst.* **2**, 2000122 (2020).
43. Hong, S. et al. Sensory Adaptation and Neuromorphic Phototransistors Based on $CsPb(Br_{1-x}I_x)_3$ Perovskite and MoS_2 Hybrid Structure. *ACS Nano.* **14**, 9796-9806 (2020).
44. Yang, H. et al. Interfacial engineering of two-dimensional metal-organic framework thin films for biomimetic photoadaptative sensors. *Chem. Mater.* **35**, 7144–7153 (2023).
45. Liao, F. et al. Bioinspired in-sensor visual adaptation for accurate perception. *Nat Electron* **5**, 84-91 (2022).
46. Zhang, M. et al. An Irradiance-Adaptable Near-Infrared Vertical Heterojunction Phototransistor. *Adv. Mater.* **34**, 2205679 (2022).
47. Kwon, S. M. et al. Environment-adaptable artificial visual perception behaviors using a light-adjustable optoelectronic neuromorphic device array. *Adv. Mater.* **31**, 1906433 (2019).
48. Lee, T.J. et al. Realization of an Artificial Visual Nervous System using an Integrated Optoelectronic Device Array. *Adv. Mater.* **33**, 2105485 (2021).
49. Si E.G. et al. Inorganic electrochromic transistors as environmentally adaptable photodetectors. Nano Energy **97**, 107142 (2022).
50. Gao Z. et al. InP Quantum Dots Tailored Oxide Thin Film Phototransistor for Bioinspired Visual Adaptation. *Adv. Funct. Mater.* **33**, 2305959 (2023).
51. He, Z. et al. An organic transistor with light intensity-dependent active photoadaptation. *Nat Electron* **4**, 522-529 (2021).
52. Liu, J. et al. Mixed-Halide Perovskite Film-Based Neuromorphic Phototransistors for Mimicking Experience-History-Dependent Sensory Adaptation. *ACS Appl. Mater. Interfaces.* **13**, 47807-47816 (2021).
