# Supplementary material for: Strain-insensitive viscoelastic perovskite film for intrinsically stretchable neuromorphic vision-adaptive transistors
Source: Nat Commun. 2024 Apr 10;15:3123. doi: 10.1038/s41467-024-47532-w (PMC11006893; doi:10.1038/s41467-024-47532-w)
Supplement: Supplementary file 3 — Description of Additional Supplementary Files [file 41467_2024_47532_MOESM3_ESM.pdf]

Description of Additional Supplementary Files

Supplementary Movies

Supplementary movie 1.

The ISNVaTs maintained a quite steady current when subjected to mechanical stain and deformation dynamically in the dark state.

Supplementary movie 2.

The ISNVaTs exhibited stable adaptive behaviors up to 50% strain, including scotopic adaptation and photopic adaptation.

Supplementary movie 3.

A vision-adaptive ISNVaT pixel circuit was constructed to visualize the adaptive phenomenon, where an ISNVaT-pixel-driven LED turned from bright to dark (photopic adaptation) and from dark to bright (scotopic adaptation).

Supplementary movie 4.

The adaptive behaviors with dynamic testing curve were visualized by the vision-adaptive ISNVaT pixel circuit.

Supplementary movie 5.

The adaptive behaviors under different lights by the vision-adaptive ISNVaT pixel circuit.
